# Contamination Assessment of Heavy Metals in Agricultural Soil, in the Liwa Area (UAE)

**DOI:** 10.3390/toxics9030053

**Published:** 2021-03-10

**Authors:** Ahmed A. Al-Taani, Yousef Nazzal, Fares M. Howari, Jibran Iqbal, Nadine Bou Orm, Cijo Madathil Xavier, Alina Bărbulescu, Manish Sharma, Cristian-Stefan Dumitriu

**Affiliations:** 1 College of Natural and Health Sciences, Zayed University, Abu Dhabi 144534, United Arab Emirates; Ahmed.Al-Taani@zu.ac.ae (A.A.A.-T.); yousef.nazzal@zu.ac.ae (Y.N.); fares.howari@zu.ac.ae (F.M.H.); Jibran.Iqbal@zu.ac.ae (J.I.); nadine.bouorm@zu.ac.ae (N.B.O.); Cijo.Xavier@zu.ac.ae (C.M.X.); manish.sharma@zu.ac.ae (M.S.); 2 Department of Earth and Environmental Sciences, Yarmouk University, Irbid 21163, Jordan; 3 Transilvania University of Brasov, 5 Turnului Str., 500036 Brasov, Romania; 4 S.C. Utilnavorep S.A., 55 Aurel Vlaicu Bd., 900055 Constanta, Romania

**Keywords:** soil, agriculture, pollution indices, heavy metals, ecological risk assessment, Liwa, Abu Dhabi, UAE

## Abstract

The Liwa area is a primary food production area in the United Arab Emirates (UAE) and has intensively been used for agriculture. This study investigates the pollution levels with heavy metals in agricultural soils from the Liwa area. Thirty-two soil samples were analyzed for Mn, Zn, Cr, Ni, Cu, Pb, Cd, Co, and As. Results revealed that heavy metal levels varied in the ranges 220.02–311.21, 42.39–66.92, 43.43–71.55, 32.86–52.12, 10.29–21.70, 2.83–8.84, 0.46–0.69, 0.03–0.37 mg/kg for Mn, Zn, Cr, Ni, Cu, Pb, Cd, Co, and As, respectively. All samples presented low As concentrations with an average of 0.01 mg/kg. The variations in bulk metal contents in the soil samples were related to multiple sources, including agrochemicals, atmospheric dust containing heavy metals, and traffic-related metals. Enrichment factor analysis indicates that Cd, Ni, Zn, and Cr were highly enriched in soils, and they could originate from non-crustal sources. Based on the geo-accumulation index (I_geo_), the soil samples appeared uncontaminated with Mn, Cr, Zn, Pb, Co, As, Cu, uncontaminated to moderately contaminated with Ni and moderately contaminated with Cd. The contamination factors suggest low contamination, except for Ni, which showed moderate contamination. The average pollution load index (PLI) revealed unpolluted to low pollution of all soil samples. The ecological risk assessment (PERI) showed that all heavy metals posed a low risk, except for Cd which exhibited a high ecological risk.

## 1. Introduction

Soil pollution with toxic heavy metals gained growing attention in recent years due to increasing human activities and expanding the industrial and transportation sectors. Urbanization has also influenced soil properties not only in the surrounding areas but also at a distance. Heavy metals are mostly toxic, persistent, and bioaccumulative. They tend to accumulate in soils [1,2,3], and transmit to the plants’ parts, which action as bioaccumulators [4,5,6,7]. The ingestion of contaminated food has adverse effects on the human health, especially on children [8,9,10,11,12,13]. The USEPA [14] listed several heavy metals as hazardous air pollutants such as Pb, Co, Mn, Ni, Cd, and Cr, of which Cd, Cr, and Ni have been included in the carcinogenic category.

Chemical compounds and heavy metals may lead to degradation or loss of some soil functions and services. Heavy metal pollution is largely irreversible [15,16,17,18,19,20]. The geology, geographical characteristics, and local climate are considered the main natural factors that influence the heavy metal dispersion in the environment. Heavy metals have a long residence time because of the interactions with particular soil components [21]. The chemical forms and metal speciation are essential factors for the fate and transport of heavy metals in soils [22], that may affect the aquifer quality as well [23]. Atmospheric deposition contributes to heavy metals accumulation in soils. While they are naturally occurring elements in the earth’s crust, human activities can increase their levels and distribution in the environment and accelerate their release from natural sources. Anthropogenic sources such as industrial emissions, fuel combustion, waste management, and transport are the most important [22,24,25,26]. Different authors pointed out the contribution of mining activities to the soil pollution, especially with heavy metals [27,28,29,30].

While a large number of publications have assessed heavy metals in urban soils [17,26,31,32,33,34,35], their status and impacts on remote soils have received less attention. The presence of heavy metals in sediments [25,36], dust [36,37] and in coastal areas [38] is considered to be a good indicator of soil contamination [2,26].

In arid regions, soil contamination with heavy metals may have different sources and pathways. Arid climates with frequent dust storms can cause heavy metals dissipation at long distances [2,39]. Elevated levels of metals were found in remote areas in arid regions and have been linked to atmospheric dust containing metals [3], among others. Besides, the fossil fuel burning explains the the input of heavy metals in some remote areas [2].

Liwa is an area in the southern UAE with a major groundwater reserve. It has been used intensively for agricultural activities. The groundwater vulnerability assessment of the Liwa’s groundwater indicated that the aquifer beneath is highly vulnerable to pollution [31].

The goal of this research is to check the hypothesis of the soil possible contamination with heavy metals in the Liwa area (Abu Dhabi Emirate). For a correct estimation of the pollution degree different indicators suggested in the literature [40,41,42,43] are used, and comparisons of results are provided. The potential sources of pollution in the area are emphasized, including agricultural activities. This study could provide additional information about the potential impact and consequences on the major groundwater aquifer in the region. This research could help the policy makers sustainably regulate the agricultural activities and adopt measures for improving crop production (because this area is the leading food producer of the UAE) and protecting the groundwater aquifer.

### Description of the Study Area

**Figure 1:**
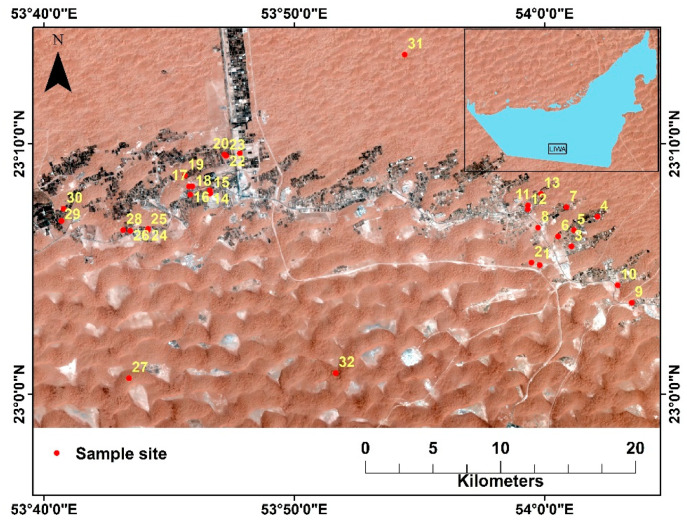
Location map of the study area and sampling sites, Liwa, Abu Dhabi.

**Figure 2:**
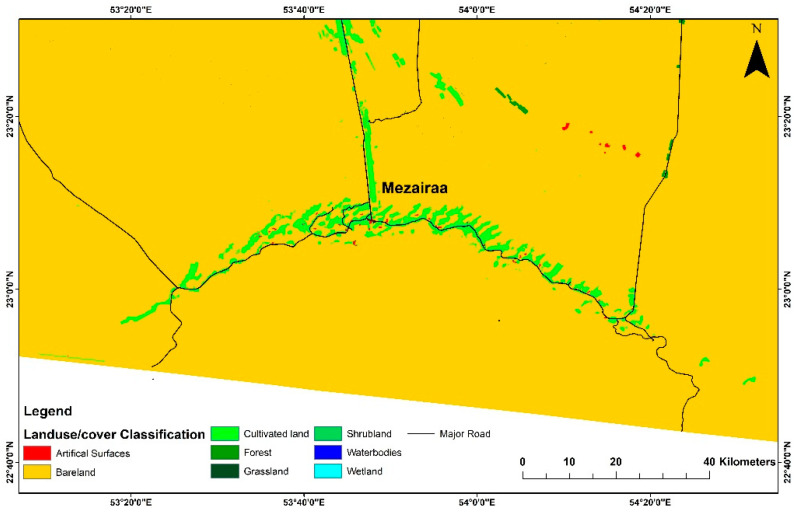
Land use/cover distribution map of the study area, Liwa, Abu Dhabi.

The study area is located in Liwa, south of Abu Dhabi, in the UAE (Figure 1). It is a sparsely populated area, where agriculture is the common land use (Figure 2).

Abu Dhabi has an arid climate with a highly variable and erratic rainfall of less than 100 mm/year, occurring mainly in winter. The annual evaporation rate in the study area is considerably high.

This area hosts a major groundwater aquifer, with a nominal groundwater recharge rate (4% of total annual water use) and no surface water resources. This shallow aquifer system in Abu Dhabi Emirate has been overused for irrigation, where the study area has one of the Emirate’s most extensive cultivated lands [44]. In addition to the intensive agriculture, desert greening has exerted additional pressure on the groundwater reserve. The main groundwater aquifers and agricultural fields are located adjacent to the main highway (between Madinat Zayed and Meziyrah), delineating the Liwa’s eastern boundary (Figure 2).

Evidence of groundwater quality degradation in the Liwa region has been reported and was attributed mainly to agricultural activities [45,46]. Shallow groundwater table and the high evaporation are other factors contributing to the deteriorating groundwater quality. Low groundwater table in this area, due to widespread irrigation, has been reported by Iqbal et al. [46].

## 2. Materials and Methods

A total of 32 soil samples were collected during February 2019 from several farming areas located in the Liwa area in UAE (Figure 1). The samples were collected from the upper 10 cm in labelled polyethylene bags. Soil samples were grinded, sieved through 2 mm mesh in the laboratory, and stored in plastic bags. 200 mg were digested in dry and clean Teflon digestion beaker, and 6 mL HNO_3_, 2 mL HCl and 2 mL HF were added, and the mixture was heated for 40 min on a hot plate at 120–150 °C. The mixture was filtered through Whatman filter paper No. 42 and the filtered digest was transferred to a 50 mL plastic volumetric flask and filled up to the mark by deionized water. Metal contents were measured by Agilent 700 series ICP-OES. A Certified Reference Material (CRM) (IAEA SOIL-7) was used to validate the analytical measurement methods. Table 1 shows the recovery results (in %) which ranges from 97–109%, indicating a high accuracy of the method used in this study.

Heavy metal pollution in soils was assessed using the Enrichment factor (EF) of Sinex and Helz [47], as follows:

EF = (M/Fe)_sample_/(M/Fe)_crust_,

where (M/Fe)_sample_ is the ratio of metal and Fe concentrations in the sample, and (M/Fe)_crust_ is the ratio of metal and Fe concentrations in the Earth’s crust [48,49]. Metals with EF < 2 are considered to originate entirely from the crustal materials or natural processes, while those with EF > 2 are most likely the product of anthropogenic activities [50].

Heavy metals in soils were also evaluated using the geoaccumulation index (I_geo_) [51]. It is expressed as:

I_geo_ = log_2_ [C*_n_*/(1.5*B_n_*)],

where *C_n_* is the measured concentration of the examined metal *n* in the soil, and *B_n_* is the geochemical background concentration (or reference value) of the metal *n*. The constant 1.5 accounts for the natural fluctuations of the metal in soil. The mean contents of the global geochemical background of Cambisols (silty and loamy soils) [49] and the average crustal abundance [52] were used. The I_geo_ has seven grades (0 to 6), indicating various degrees of enrichment above the background values and ranging from unpolluted to very highly polluted soil quality.

The contamination factor (CF) for soil samples were assessed to quantify the impact of each metal on the soil [53,54]. The CF was calculated by dividing the heavy metal concentration of soil by the background value of reported by Kabata-Pendias [49].

The pollution degree was further assessed by the pollution load index (PLI). The PLI of each metal was computed as follows:

PLI = (CF_1_ × CF_2_ × … × CF_n_) ^1/n^,

where CF is the calculated contamination factor, *n* is the number of heavy metals (nine in the present study) [55,56,57]. The PLI of each metal was classified as polluted (PLI > 1), or within the baseline level (PLI = 1), whereas PLI < 1 indicates no pollution [56].

The potential ecological risk index was computed to assess the cumulative pollution effects of multiple metal on the soils. The formula developed by Håkanson [54] was used, as follows:

PERI = ∑E_i_,

E_i_ = T_i_ × CF_i_,

where E_i_ is the single ecological risk index, T_i_ is the toxic response factor for a given metal, *i* (e.g., Cd = 30, Mn = Zn = 1, Pb = Ni = Co = Cu = 5, As = 10, and Cr = 2) [51], CF_i_ is the calculated contamination factor for the metal *I*, E_i_ < 40 indicates low contamination, whereas 40 ≤ E_i_ < 80, 80 ≤ E_i_ < 160, 160 ≤ E_i_ < 320, and E_i_ ≥ 320 shows a moderate, considerable, high, or significantly high contamination risk, respectively [54] and CF_i_ indicates a low (CF_i_ < 1), moderate (1 ≤ CF_i_ < 3), considerable (3 ≤ CF_i_ < 6), or very high contamination (CF_i_ ≥ 6) [54].

The PERI has four grades, corresponding to values smaller than 150 (low), from 150–300 (moderate), in the range of 300–600 (considerable), and greater than or equal to 600 (very high) [54].

## 3. Results and Discussion

The summary of the statistical analysis of heavy metal concentrations in the soil samples is presented in Table 2. Heavy metal levels varied from 220.02–311.21, 42.39–66.92, 43.43–71.55, 32.86–52.12, 10.29–21.70, 2.83–8.84, 0.46–0.69, 0.03–0.37 for Mn, Zn, Cr, Ni, Cu, Pb, Cd, and Co mg/kg, respectively. The metals concentrations are slightly variable, with the highest variability observed for Co followed by Pb (with coefficients of variation of 0.45 and 0.32, respectively). Low levels were observed in all soil samples averaging of 0.01 mg/kg) (Table 2).

**Figure 3:**
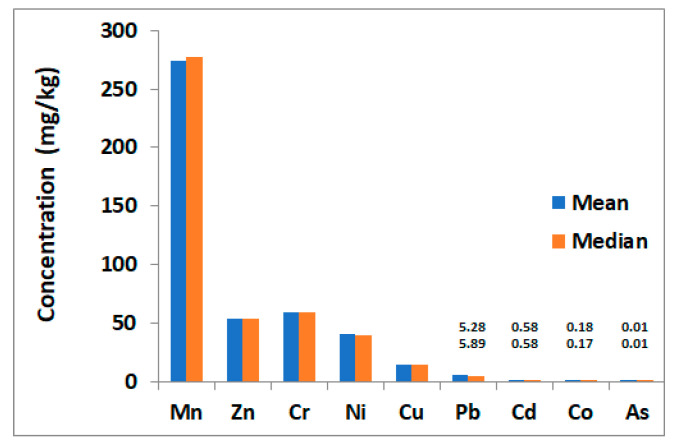
Mean and median concentrations (mg/kg) of heavy metals in the soil samples collected from Liwa, Abu Dhabi.

The highest concentration was that of Mn, with mean and median concentrations of 273.9 and 277.22 mg/kg, respectively. The concentrations of the other heavy metals, in decreasing order, are Cr > Zn > Ni > Cu > Pb > Cd > Co > As (Figure 3 and Table 2).

The variations in concentrations of bulk metals in the soil samples are related to multiple sources. This region hosts the largest groundwater aquifer in the UAE and has intensively been used for agricultural production (Figure 2). Agrochemicals may contribute to heavy metals accumulation in soils (such as Cu, Zn, Fe, Mn, and As) [22]. Elevated levels of Cd, As, and Pb have been associated with applications of phosphate fertilizers [58].

The compost is frequently used in the UAE to enhance the soil properties and improve its fertility since infertile sandy soils are widespread in the UAE. The use of compost (and other biosolids such as livestock manure and municipal sewage sludge) have been reported to increase the concentration of certain metals (As, Cd, Cr, Cu, Pb, Hg, Ni, Se, Mo, Zn, Tl, Sb, etc.) in soils [59]. These metals can be released from the compost and other biosolids to the soils and can end up in the groundwater aquifers [60,61,62,63]. Enriched soil with metals (such as Cu, Zn, Ni, Pb, Cd, and Cr), as a result of repeated application of biosolids, has been reported [64,65]. The frequent use of fungicides and pesticides has also been associated with the accumulation of heavy metals (such as Cu, Zn, Pb, and As) in soils [64,65,66,67].

**Figure 4:**
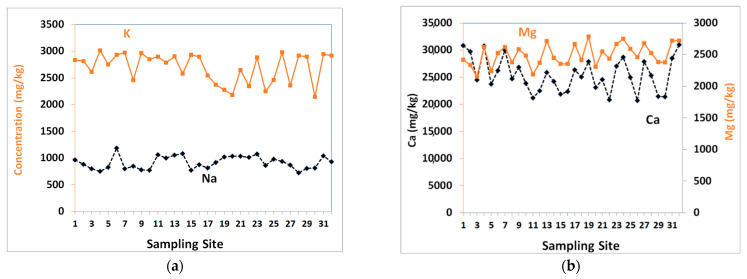
The concentrations of (**a**) Na, and K, (**b**) Ca and Mg in the soil samples collected from Liwa.

Intensive agricultural production is commonly practiced in this region, where groundwater is the sole source of irrigation water. Salinization and contamination with chemicals were observed in groundwater [39,46]. The soils’ compositions revealed greater concentrations of Ca, Mg, Na and K (Figure 4) that are attributed to overirrigation with salty groundwater, weathering of carbonates and evaporites rocks that are widespread in this region.

The Liwa region’s vulnerability assessment indicated that the aquifer beneath the extensive agriculture area is highly vulnerable to pollution [39]. It indicates that heavy metals in soils may leach with agricultural runoff to end up in the groundwater.

The study area is also bordered by a major traffic highway, with impacts from vehicular emissions. Cd, Pb, and Zn in soils could originate partly from traffic activities [24,68,69,70]. Elevated levels of heavy metals were observed in roadside dust samples collected from Abu Dhabi, particularly Cr, Pb, Zn, and Mn [2] (Table 3). However, sediment samples collected from the coastal area of Abu Dhabi further from the major highway showed low concentration [71] (Table 3). This suggests that ambient dust is probably an important source and pathway of metals to the soil in Liwa.

Heavy combustion of fossil fuel is another potential source contributing to heavy metals’ measured values in soil (especially Cd, Zn, As, Cu, Mn), where the study area is located near gas and oil fields in the southern UAE. Also, the Arabian Peninsula (including the UAE) hosts leading oil and petrochemical industries. These world’s industries can add metals to the ambient dust, where the northerly wind carries airborne emissions to the UAE [2,3].

The electric power industry is the backbone of the UAE economic sectors and is expanding rapidly. The UAE’s energy consumption rate is one of the highest globally [72]. Natural gas-fueled power plants are essential contributors to the metal concentration in the ambient air, and consequently, to the measured values of heavy metals in soil, through dust deposition. Heavy oil and diesel are occasionally used in power plants [73].

Dust storms are common in the UAE, contributing to the transport and deposition of the metals to soil [2,11]. The UAE is in a desert-belt region with intense and frequent dust storm events, where the surrounding deserts are the major sources of mineral dust-containing heavy metals [2,3]. Regional deserts (in Iran, Pakistan) are likely to contribute to long-range atmospheric dust in the UAE [74].

While the composition of soils revealed generally much greater concentrations for Mn, Zn, Cr, and Ni than other metals, only the concentration of Cd and Ni in the soil samples exceeded the background levels for the continental crust and the average worldwide soils. In contrast, the remaining heavy metals were within the reported limits (Table 3). Heavy metals concentrations in soils are higher than those observed in the coastal sediments of Abu Dhabi but lower than those for roadside dust (except for Ni and Cd) (Table 3).

**Figure 5:**
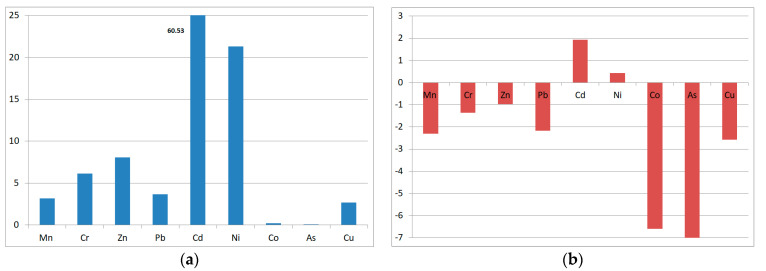
The average (**a**) geo-accumulation index (I_geo_), (**b**) enrichment factors (EF)relative to the average upper continental crust for heavy metals in the soil samples collected from Liwa, Abu Dhabi, UAE.

Enrichment factor (EF) analysis showed that Cd was the most enriched element followed by Ni, Zn, Cr, Mn, Co and As (Figure 5).

According to the enrichment classification [75], the low EF levels for As (0.06) and Co (0.19) suggest that they are entirely originated from the crustal materials or natural processes, as they may release during the weathering and erosion of igneous rocks. These metals are transported and deposited by atmospheric mineral dust due to frequent and active dust storms. However, Cd (60.53), Ni (21.31), Zn (8.06), and Cr (6.16) are highly enriched in soils. They could originate from non-crustal sources. The use of phosphoric fertilizers can add Cd and Pb to the soil [76] and the vehicles’ exhaust as the area is surrounded by major highways. Pb (3.67) and Mn (3.18) are moderately enriched and mainly derived from various sources, with the natural one being the major contributor. Pb can be attributed to the vehicle exhausts (because the leaded gasoline is used for trucks loading/unloading) and the dry deposition.

Zn can be associated with vehicular emissions such as combustion of petrol, wearing of tires and brake linings. In addition to anthropogenic sources, Cr content values in soil samples (compared to world soil average content of Cr) also suggest that Cr is derived from the chemical weathering of basalt exposed in the region and carried through atmospheric dust. Cr concentration of about 170 mg/kg was measured in basalt [77], whereas the average crustal abundance is 100 mg/kg [49]. The relative low Cu content in soil indicates natural sources, with a minor contribution of anthropogenic sources such as fungicide and pesticides enriched in Cu [78]. High concentrations of Ni were observed in soil samples that might be related to the fuel additive [79], where diesel-powered trucks are loading/unloading materials (crops, fertilizers, etc.), among other sources already described.

**Figure 6:**
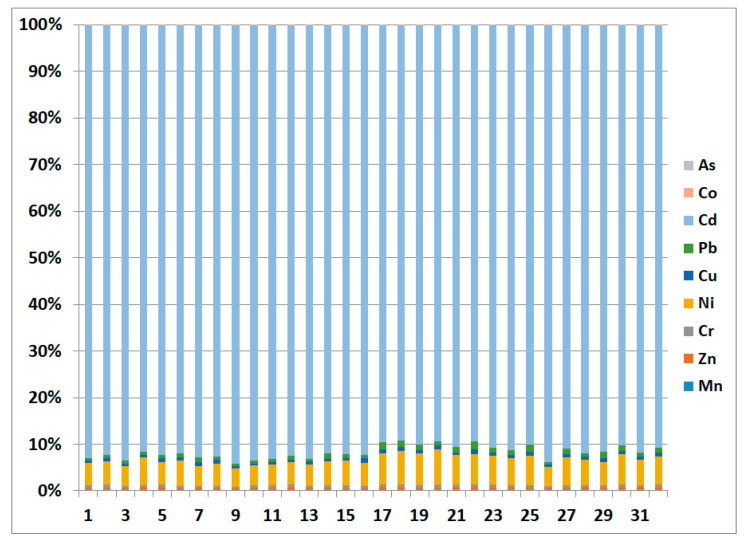
Contribution of heavy metals to the E_i_ in soil samples, Liwa, UAE.

Based on Muller scales [51], the soil samples are uncontaminated with Mn, Cr, Zn, Pb, Co, As, Cu (with I_geo_ of −2.31, −1.36, −0.97, −2.17, −6.59, −8.14 and −2.57). Soils are uncontaminated to moderately contaminated with Ni (I_geo_ = 0.43) and moderately contaminated with Cd (I_geo_ = 1.94) (Figure 6 and Table 4). Heavy metals are ranked in the following order (based on I_geo_): Cd > Ni > Zn > Cr > Pb > Mn > Cu > Co > As (Figure 6 and Table 4).

The CF of metals in soil samples (Table 4) indicates low contamination, except for Ni, which shows moderate contamination. Based on the CF, heavy metals are ranked in the following order: Cd > Ni > Zn > Cr > Pb > Mn > Cu > Co > As.

The average PLI values indicated unpolluted to slightly polluted soils samples (Table 4). The results of PERI of heavy metals in the soil (Table 4) revealed that the single ecological risks (E_i_) of heavy metals were ranked in the following order: Cd > Ni > Pb > Cu > Cr > Zn > Mn > Co > As. All heavy metals have E_i_ values indicating low risk, except for Cd, which possesses a high ecological risk. The PERI in soils ranged from 152.63 to 224.39 with an average of 189.28 (Figure 5). These values indicate a moderate potential ecological risk [54].

The percent contribution of individual metal to overall PERI is shown in Figure 6. It relevels that Cd accounts for approximately 91.6% of the total ecological risk, whereas Ni represents about 5.6%, and the remaining heavy metals compromise 2.8% only. These results indicate that Cd primarily pose the high ecological risk as they contribute 97.2% to the total potential ecological risk in the soils.

The high variation coefficients of certain heavy metals (such as Pb and Co) showed values larger than 30%, suggesting that they were probably derived from different emission sources. These metals probably originated from multiple sources, of which some are common (as discussed earlier). Some metals are released from natural sources and enriched by anthropogenic activities, such as traffic-related emissions, agriculture, and fuel combustion.

## 4. Conclusions

This study provides valuable data about heavy metal contents in soils from Liwa (Abu Dhabi). Liwa is of prime importance as it is a major food production area of the country. A previous study had shown that the major groundwater aquifer located beneath the extensive agriculture area lies within high to very high pollution vulnerability zones. This also indicates that heavy metals in soils may leach with agricultural runoff to end up in groundwater. The composition of soils revealed generally much greater concentrations for Mn, Zn, Cr, and Ni compared to other metals, but only the concentration of Cd in soil samples exceed the background levels for the continental crust and the average worldwide soils. Even if Cd, Ni, Zn, and Cr are highly enriched in soils, the pollution indices generally showed that the soils are uncontaminated with the majority of heavy metals except for Ni and Cd. The ecological risk assessment revealed a low risk to local ecosystems, except for Cd.

## Figures and Tables

**Table 1:** The accuracy and precision of the analytical method applied to the multi-element determination of soil samples.

Element	Mass	Certified Value (mg/Kg)	Measured Value (mg/Kg) *	Recovery (%)
Mn	55	631	641.1	101.6
Zn	66	104	101.94	98.02
Cr	52	60	61.71	102.85
Ni	60	26	26.76	102.92
Cu	63	11	10.82	98.36
Pb	208	60	61.6	102.67
Cd	111	1.3	1.31	100.77
Co	59	8.9	9.76	109.66
As	75	13.4	14.01	104.01

* The mean of five consecutive samples.

**Table 2:** Metal concentrations (mg/kg) in the soil samples collected from Liwa, Abu Dhabi. SD is the standard deviation and CV is the coefficient of variation.

	Mean	Min	Max	SD	Median	CV
Mn	273.9	220.02	311.21	21.49	277.22	0.08
Zn	54.08	42.39	66.92	7.47	54.07	0.14
Cr	59	43.43	71.55	7.85	58.5	0.13
Ni	40.83	32.86	52.12	5.74	39.24	0.14
Cu	14.17	10.29	21.7	2.68	14.07	0.19
Pb	5.28	2.83	8.84	1.72	4.89	0.32
Cd	0.58	0.46	0.69	0.06	0.58	0.11
Co	0.18	0.03	0.37	0.08	0.17	0.45
As	0.016	0.01	0.01	0.01	0.01	0.06

**Table 3:** Heavy metals concentration (mg/kg) in this study compared to their averages in the upper continental crust, worldwide soils, and coastal sediments and roadside dust in Abu Dhabi.

	Present Study	Continental Crust [48,49]	Worldwide Soils [49]	Abu Dhabi Coastal Sediment [71]	Abu Dhabi Roadside Dust [2]
As	0.01	1.8	6.83	1	0.23
Cd	0.58	0.1	0.41	0	0.48
Cr	59	100	59.5	-	306.3
Co	0.18	10	11.3	4.1	-
Cu	14.17	55	38.9	3.8	-
Pb	5.28	15	27	1.9	50.05
Ni	40.83	20	29	25.3	0.3
Zn	54.08	70	70	8.2	173.0
Mn	273.9	900	488	-	1158.5

**Table 4:** Basic statistics of the contamination factor (CF), pollution load index (PLI), single ecological risk index (E_i_), and PERI for heavy metals in the soils from Liwa, Abu Dhabi.

	**CF**									**PLI**
	**Mn**	**Zn**	**Cr**	**Ni**	**Cu**	**Pb**	**Cd**	**Co**	**As**	
Mean	0.30	0.77	0.59	2.04	0.26	0.35	5.79	0.02	0.01	0.29
Min	0.24	0.61	0.43	1.64	0.19	0.19	4.61	0.00	0.00	0.19
Max	0.35	0.96	0.72	2.61	0.39	0.59	6.92	0.04	0.01	0.39
SD	0.03	0.11	0.08	0.30	0.05	0.12	0.65	0.01	0.00	0.04
	**E_i_**									**PERI**
Mean	0.30	0.77	1.18	10.21	1.29	1.76	173.63	0.09	0.05	189.28
Min	0.24	0.61	0.87	8.22	0.94	0.94	138.31	0.02	0.05	152.63
Max	0.35	0.96	1.43	13.03	1.97	2.95	207.63	0.18	0.06	224.39
SD	0.02	0.11	0.16	1.44	0.24	0.57	18.60	0.04	0.00	18.26
	**I_geo_**									
Mean	−2.31	−0.97	−1.36	0.43	−2.57	−2.17	1.94	−6.59	−8.14	
Min	−2.62	−1.31	−1.79	0.13	−3.00	−2.99	1.62	−8.75	−8.29	
Max	−2.12	−0.65	−1.07	0.80	−1.93	−1.35	2.21	−5.34	−7.88	
SD	0.12	0.20	0.20	0.20	0.26	0.46	0.16	0.88	0.09	

## Data Availability

The raw data supporting the conclusions of this article will be made available by the authors, without undue reservation.
